# Prediction of catheter navigation difficulty during mechanical thrombectomy using CTA-based carotid siphon morphometry

**DOI:** 10.3389/fmedt.2026.1900255

**Published:** 2026-07-29

**Authors:** Ferenc Dezső Bakó, Attila Tanács, József Tolnai, Pál Barzó, Tamás Németh, Attila Nagy

**Affiliations:** 1 Department of Medical Physics and Informatics, University of Szeged, Szeged, Hungary; 2 Department of Image Processing and Computer Graphics, Institute of Informatics, University of Szeged, Szeged, Hungary; 3 Department of Neurosurgery, University of Szeged, Szeged, Hungary

**Keywords:** aspiration catheter, carotid siphon, CT angiography, device escalation, machine learning, mechanical thrombectomy, morphometry

## Abstract

**Introduction:**

Successful catheter navigation through the carotid siphon remains a major technical challenge during mechanical thrombectomy for acute ischemic stroke. Unfavorable vascular anatomy may require stepwise device escalation, prolonging procedural time and potentially delaying reperfusion. Because conventional two-dimensional measurements may not fully capture the three-dimensional vascular features influencing device behavior, this study evaluated whether CTA-derived carotid siphon morphometry can predict the level of device support required for aspiration catheter navigation.

**Methods:**

This retrospective study included patients who underwent endovascular thrombectomy at the Department of Neurosurgery, University of Szeged, between 15 April 2020 and 16 May 2022. From an initial cohort of 160 patients, 54 CTA datasets were suitable for three-dimensional segmentation, and 53 cases were included in the final morphometric analysis. Paired native CT and CTA scans were processed using a subtraction-based workflow in 3D Slicer, followed by vesselness filtering, segmentation, and centerline extraction. Local carotid siphon features, including cross-sectional area and curvature-related distance measurements, were extracted. Procedural difficulty was defined using a stepwise device-escalation scale reflecting the support required to advance the aspiration catheter through the carotid siphon. A logistic regression classifier with ridge regularization and correlation-based feature selection was trained using nine geometric attributes.

**Results:**

In the full 53-case dataset, the model achieved 58.5% accuracy, with substantial interclass overlap, particularly among higher-complexity categories. Applying a confidence-based decision criterion, retaining only predictions where the second-highest class probability was less than 60% of the highest class probability, yielded a high-confidence subset of 32 cases. In this subset, accuracy increased to 75.0%, the kappa statistic increased from 0.40 to 0.65, and the weighted F-measure reached 0.73. The model achieved 100% recall for M2 and 100% precision for M3, whereas M4 remained difficult to distinguish.

**Conclusion:**

CTA-derived three-dimensional morphometric analysis of the carotid siphon provides clinically relevant information on catheter navigation difficulty during mechanical thrombectomy. Confidence-gated classification identified anatomically well-defined cases in which device-support requirements could be predicted more reliably. Borderline cases remained influenced by factors beyond luminal geometry. Larger multicenter studies are warranted to validate this approach and assess its role in patient-specific preprocedural planning.

## Introduction

Mechanical thrombectomy has become the standard of care for acute ischemic stroke caused by large-vessel occlusion (1–3). Several endovascular strategies are currently used in clinical practice, including direct aspiration, stent retriever-based thrombectomy, and combined approaches that integrate both techniques. In direct aspiration, a large-bore catheter is advanced to the thrombus face and continuous suction is applied to achieve clot removal. In contrast, stent retrievers engage and extract the thrombus via a self-expanding device, while combined techniques aim to maximize first-pass reperfusion by leveraging the advantages of both approaches (4–6).

Randomized clinical trials and comparative studies have demonstrated broadly comparable efficacy among these techniques (5–7). Notably, the ASTER trial showed no clear superiority of first-line contact aspiration over stent retriever thrombectomy in terms of functional outcomes or recanalization rates (8). However, aspiration-based strategies may offer procedural advantages in selected cases, including shorter procedure times and reduced device exchanges (4, 6). This has been further supported by recent prospective multicenter data, such as the SESAME study, which demonstrated the safety and effectiveness of large-bore aspiration catheters as first-line devices in anterior circulation stroke (9). In favorable anatomical conditions, direct catheter navigation, occasionally even without a leading microwire, may simplify the procedure and reduce the number of technical steps (4, 10).

Despite continuous advances in device technology, successful catheter navigation through the carotid siphon remains a critical technical challenge in mechanical thrombectomy (11–13). The carotid siphon represents a highly tortuous and anatomically complex vascular segment, and unfavorable geometry may lead to procedural delays, repeated device escalation, or even failure to achieve intracranial access. Given that time to reperfusion is a key determinant of neurological outcome, such delays may have direct clinical consequences (3, 14, 15). However, the anatomical determinants of catheter navigation difficulty in this region remain incompletely understood.

Previous studies have characterized carotid siphon anatomy using simplified geometric descriptors derived from angiographic or cross-sectional imaging. Waihrich et al. examined the relationship between carotid siphon anatomy and aneurysm presentation, Sugiyama et al. evaluated the influence of internal carotid artery angulation on endovascular treatment of paraclinoid aneurysms, and Labeyrie et al. assessed carotid siphon morphology in relation to posterior communicating artery aneurysms (16–18). These studies demonstrate the clinical relevance of carotid siphon geometry, but they primarily address aneurysm-related morphology and rely on simplified angular or anatomical descriptors. Such reduced representations may not fully capture the three-dimensional geometric complexity that influences catheter behavior during mechanical thrombectomy.

More recently, three-dimensional modeling approaches have been proposed to address these limitations. Bridio et al. demonstrated, using virtual thrombectomy simulations, that specific curvature characteristics of the carotid siphon, particularly the anterior bend, may influence procedural success (19). Although this work represents an important conceptual advance, it was based on a limited sample size and has not yet been validated in clinical practice.

Taken together, these findings highlight the need for more comprehensive, three-dimensional approaches to the assessment of carotid siphon anatomy. In particular, CTA-based geometric analysis may offer a clinically accessible means of quantifying vascular complexity and predicting procedural difficulty. Accurate preprocedural estimation of catheter navigability could help optimize device selection, streamline procedural workflow, reduce time to reperfusion, and minimize unnecessary device escalation.

The aim of the present study was to evaluate whether image- and geometry-based features extracted from three-dimensionally reconstructed CTA datasets of the carotid siphon can predict the level of device support required for successful catheter navigation through the carotid siphon during mechanical thrombectomy. Because the investigated endpoint was catheter advancement to the thrombus rather than thrombus retrieval itself, thrombus location, size, and composition were not included in the present analysis. By correlating quantitative morphometric parameters of the carotid siphon with actual procedural device use, we sought to assess whether CTA-derived anatomical information could serve as a foundation for future decision-support strategies in endovascular stroke treatment.

## Materials and methods

### Participants

The study protocol was approved by the Human Investigation Review Board of the University of Szeged, Hungary (Approval No. 91/2022-SZTE-RKEB; Date: 23 May 2022). The study was conducted in accordance with the Declaration of Helsinki and its subsequent amendments.

### Inclusion criteria

Patients were screened for inclusion if they underwent endovascular thrombectomy (EVT) for acute ischemic stroke at the Department of Neurosurgery, University of Szeged, between 15 April 2020 and 16 May 2022.

Patient selection for EVT followed contemporary clinical practice based on major randomized trials and current international stroke guidelines. In general, patients presenting within 6 h of symptom onset were considered eligible if they were aged ≥18 years, had a National Institutes of Health Stroke Scale (NIHSS) score ≥6, and had good pre-stroke functional status, defined as a modified Rankin Scale score of 0–2. Baseline non-contrast CT was used to exclude extensive early ischemic changes, while CT angiography (CTA) was performed to confirm large-vessel occlusion of the middle cerebral artery (MCA), involving the M1 or proximal M2 segment.

For patients presenting beyond the conventional 6-hour time window, additional imaging with CT perfusion was used to assess infarct core size and the presence of salvageable brain tissue, based on established clinical-core mismatch or target mismatch criteria derived from landmark trials. After application of the initial clinical criteria, 160 EVT cases were identified for imaging review. Following imaging, anatomical, and technical exclusions, 54 CTA datasets underwent three-dimensional segmentation, and 53 cases were included in the final morphometric analysis.

### Exclusion criteria

Patients were excluded if preprocedural CTA data were unavailable, including cases in which imaging had been performed at referring institutions and could not be retrieved for analysis (*n* = 50). Among cases with available CTA imaging, further exclusions were applied for posterior circulation occlusions (*n* = 25), or occlusions involving the carotid siphon or more proximal arterial segments (*n* = 20), carotid bifurcation occlusions (*n* = 2), unsuccessful catheter navigation through the carotid siphon (*n* = 5), and CTA datasets with insufficient intraluminal contrast enhancement precluding reliable three-dimensional reconstruction and geometric segmentation (*n* = 4).

**Figure 1:**
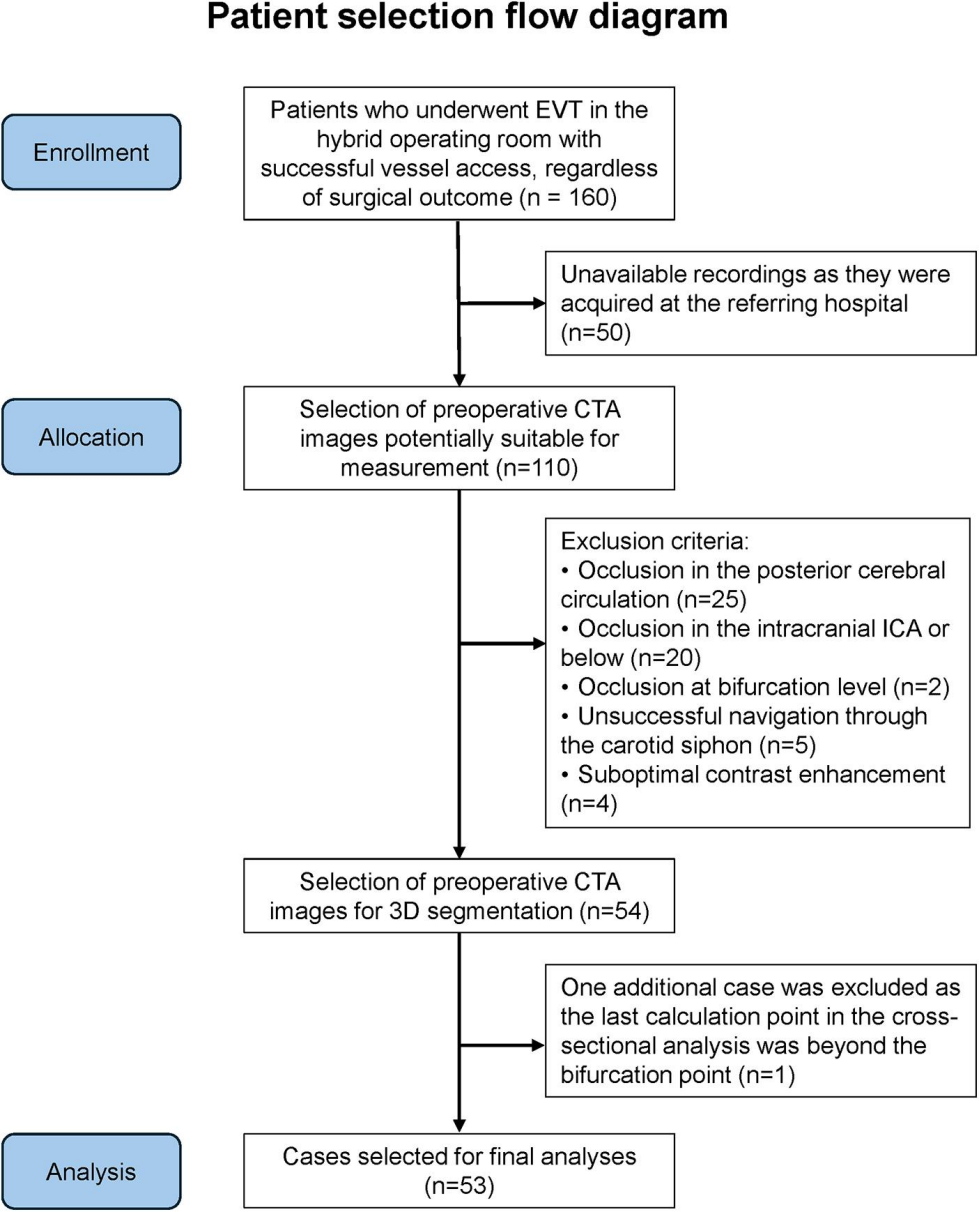
Patient selection flow diagram. Flowchart illustrating the stepwise selection of patients included in the study. A total of 160 patients underwent endovascular thrombectomy (EVT) with successful vessel access. Preprocedural CTA datasets were unavailable in 50 cases, leaving 110 patients with potentially suitable imaging. Further exclusions were applied due to posterior circulation occlusions (*n* = 25), intracranial internal carotid artery or distal occlusions (*n* = 20), bifurcation-level occlusions (*n* = 2), unsuccessful navigation through the carotid siphon (*n* = 5), and insufficient contrast enhancement (*n* = 4). CTA-based three-dimensional segmentation was performed in 54 cases. One additional case was excluded from cross-sectional analysis due to the distal measurement point extending beyond the carotid bifurcation. The final study cohort consisted of 53 patients.

Following three-dimensional segmentation, one additional case was excluded from cross-sectional analysis because the distal measurement point extended beyond the carotid bifurcation (*n* = 1). The study was reported in accordance with STROBE guidelines, and the patient flow diagram is presented in Figure 1.

### Baseline demographic and clinical characteristics

A summary of baseline demographic and clinical characteristics of the study population is provided in Table 1.

**Table 1:** Baseline demographic and clinical characteristics of the study population.

Variable	Mean ± SD	Median	Range	*n* (%)
Age (years)	74.3 ± 11.1	75	47–97	
NIHSS at admission	13.9 ± 6.5	13	3–42	
eASPECTS score	8.6 ± 1.2	9	6–10	
Female sex				30 (57%)
Atrial fibrillation (known or newly diagnosed)				24 (45%)
Hypertension (known or newly diagnosed)				44 (83%)
Diabetes mellitus (known or newly diagnosed)				11 (21%)
Intravenous thrombolysis				14 (26%)
Successful reperfusion (mTICI ≥ 2b)				28 (53%)
Postprocedural haemorrhage				2 (4%)
mRS at 30 days	3.5 ± 2.2	4	0–6	
mRS at 90 days	3.9 ± 2.3	5	0–6	

Continuous variables are presented as mean ± standard deviation (SD), median, and range. Categorical variables are presented as absolute numbers and percentages.

### Procedural technique and device escalation scale

Mechanical thrombectomy procedures performed during the study period followed a standardized stepwise escalation protocol at the Department of Neurosurgery, University of Szeged. For each case, the level of device support required to advance the aspiration catheter through the carotid siphon was recorded.

This procedural classification reflects the increasing complexity and structural support of the endovascular system required to overcome vascular tortuosity or anatomical resistance during catheter navigation to the site of occlusion. We hypothesized that the requirement for higher-level device assistance serves as a surrogate marker of procedural difficulty and reflects the challenges imposed by patient-specific vascular anatomy.

Procedural escalation was categorized into five levels. In Method 0 (M0, direct aspiration, ADAPT), the large-bore aspiration catheter was advanced directly to the thrombus without the use of a leading microwire or microcatheter. This configuration represents the most favorable vascular anatomy, in which the catheter can traverse the carotid siphon without additional support.

In Method 1 (M1, microwire-assisted aspiration), a steerable microwire, typically 0.014″ in diameter, was used to guide the aspiration catheter through the carotid siphon, while the aspiration catheter remains the primary device for distal access.

Method 2 (M2, standard coaxial/triaxial navigation) involved advancing a microcatheter over the microwire to establish a stable rail for aspiration catheter delivery. This coaxial or triaxial configuration provided improved support for navigating the curved segments of the carotid siphon.

In Method 3 (M3, reinforced high-support navigation), high-support (“extra-support”) microwires were used in cases of pronounced vascular tortuosity to increase system stability and prevent catheter prolapse during advancement through the cavernous segment of the internal carotid artery.

Method 4 (M4, stent retriever-assisted navigation, anchor technique) represented the highest level of procedural escalation. In this configuration, a stent retriever was deployed distal to the occlusion to serve as a stationary anchor, enabling forceful advancement of the aspiration catheter through the carotid siphon when all preceding navigation strategies had failed.

These procedural categories are summarized in Table 2.

**Table 2:** Procedural device-escalation scale for classification of carotid siphon navigation difficulty.

Method	Navigation strategy	Devices required	Interpretation of anatomical difficulty
0	Direct aspiration (ADAPT)	Aspiration catheter only	Very favorable anatomy, catheter passes siphon without assistance
1	Microwire-assisted aspiration	Aspiration catheter + microwire	Mild anatomical resistance requiring wire guidance
2	Standard coaxial/triaxial navigation	Aspiration catheter + microwire + microcatheter	Moderate tortuosity requiring a stable coaxial support system
3	Reinforced high-support navigation	Aspiration catheter + extra-support microwire ± microcatheter	Severe tortuosity requiring increased system stiffness
4	Stent retriever–assisted navigation (anchor technique)	Aspiration catheter + stent retriever anchor system	Extreme anatomical difficulty requiring distal anchoring

### CTA recordings and segmentation

Cranial CT and CT angiography (CTA) images were used for analysis. Each case included paired native and contrast-enhanced acquisitions performed on a GE Revolution EVO scanner (120 kV tube voltage, 121*–*400 mA tube current, generator power approximately 40*–*48 kW). Images were reconstructed using a 512 × 512 matrix with an in-plane spatial resolution of approximately 0.45 mm and slice thicknesses ranging from 0.625 to 0.762 mm. The iodinated contrast agent Omnipaque 350 (iohexol) was administered for angiographic acquisition.

A total of 54 subjects were initially included, each represented by paired native and angiographic scans, resulting in 108 imaging series. A summary of imaging parameters is provided in Table 3.

**Table 3:** Summary of CT imaging parameters used for vessel segmentation.

Parameter	Value/range
Scanner model	Revolution EVO (GE healthcare)
Tube voltage (kV)	120 kV (mean ≈ 119 kV)
Tube current (mA)	121–400
Exposure time (ms)	500–1,460
Matrix size	512 × 512
Mean voxel size (mm)	0.449 × 0.449 × 0.762
Slice thickness (mm)	0.625–0.762
Contrast agent	Omnipaque 350 (iohexol)
Number of subjects	53
Total number of series	106

**Figure 2:**
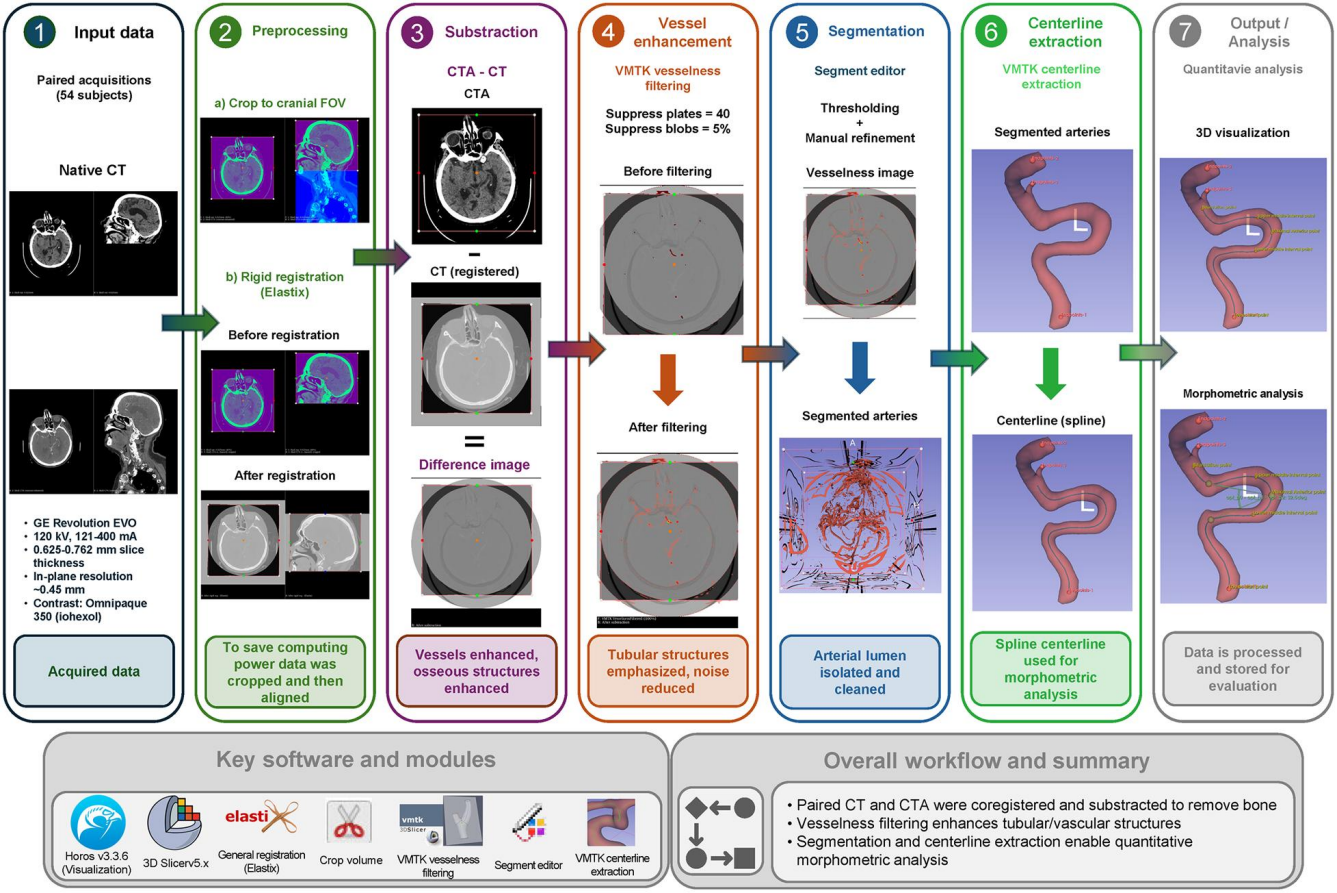
CT/CTA image-processing and segmentation workflow. Stepwise overview of the image-processing pipeline used to obtain carotid siphon segmentations and centerlines for morphometric analysis. Paired native CT and CTA datasets were first cropped and coregistered. Voxel-wise subtraction was then performed to suppress osseous structures and enhance contrast-filled vessels. Vascular structures were further emphasized using VMTK vesselness filtering, followed by threshold-based segmentation and manual refinement in 3D Slicer. Cleaned arterial segmentations were used for VMTK centerline extraction, and the resulting spline centerlines served as the basis for subsequent quantitative morphometric analysis. Horos, 3D Slicer, Elastix, Segment Editor, and VMTK modules were used during the workflow.

Image analysis and segmentation were performed using the open-source software Horos v3.3.6 and 3D Slicer v5.2.2 (20). Segmentation of intracranial arteries was complicated by the similar radiodensity of osseous structures and contrast-enhanced vessels. To address this issue, a subtraction-based segmentation workflow was applied according to the Vessel Segmentation by Subtraction method described by Lasso et al. (21) By subtracting the native CT from the corresponding CTA dataset, a residual image emphasizing contrast-filled vascular structures was obtained, thereby facilitating arterial segmentation. The complete CT/CTA preprocessing, subtraction, vessel enhancement, segmentation, and centerline extraction workflow is illustrated in Figure 2.

Because even minor patient motion between acquisitions could degrade subtraction accuracy, precise image coregistration was essential. Rigid registration between native and angiographic datasets was performed using the General Registration (Elastix) module of 3D Slicer. In cases where automatic registration failed due to excessive displacement, anatomical landmarks were manually aligned using the Transform module before repeated registration. Prior to registration, angiographic datasets were cropped using the Crop Volume module to match the field of view of the native cranial CT, thereby reducing unnecessary anatomical information and improving registration convergence. Alignment quality was visually verified by simultaneously displaying the datasets in contrasting colors using the Volumes module.

Following successful registration, voxel-wise subtraction generated a difference image in which vascular structures remained while osseous tissues were largely suppressed. Vessel enhancement was further improved using the Vascular Modeling Toolkit (VMTK) Vesselness Filtering module (22, 23), with optimal delineation achieved at Suppress Plates = 40% and Suppress Blobs = 5%. Segmentation was then performed using the Segment Editor module of 3D Slicer. Threshold values were manually adjusted to isolate the arterial lumen and refined interactively where necessary. Although subtraction and filtering occasionally introduced minor artifacts, manual correction proved substantially faster than slice-by-slice segmentation.

Finally, vascular centerlines were extracted from the cleaned segmentations using the VMTK Centerline Extraction module. The resulting spline curves served as the basis for quantitative morphometric analysis.

Imaging metadata were extracted from DICOM headers and compiled into a structured database for quality assurance and evaluation of acquisition parameters. All datasets were anonymized prior to analysis.

### Three-dimensional morphological model and carotid siphon parameters

The carotid siphon represents the most anatomically challenging segment for aspiration catheter navigation, and it was therefore assumed that catheter advancement may be hindered primarily by the curvature and caliber of this vascular region. Following segmentation and centerline extraction, geometric features describing vessel morphology were quantified and correlated with procedural device-escalation categories.

The analysis pipeline consisted of four principal steps: (1) spatial normalization by determining the center point of the carotid siphon curvature, (2) sampling of geometric features around this reference point, (3) training of classification models using the extracted geometric parameters and procedural ground-truth labels, and (4) prediction of the most probable intervention scenario based on vascular morphology.

A total of 53 studies were available for final analysis. Because Method 0 contained only two cases after application of the inclusion and exclusion criteria, reliable statistical learning of this class was not feasible. Furthermore, M0 and M1 represent anatomically similar low-complexity navigation scenarios differing only in whether microwire assistance was required. Therefore, the original five intervention classes were reduced to four for classification analysis. Consequently, the model predicts a single low-complexity navigation category rather than distinguishing between M0 and M1, a simplification that has minimal practical impact because these two categories represent clinically similar procedural scenarios. Given the limited sample size relative to the number of procedural categories, the number of geometric features included in the model was intentionally restricted to reduce overfitting.

Two principal geometric properties were analyzed: vessel cross-sectional area and vessel curvature. Cross-sectional area was determined by constructing a plane perpendicular to the vessel centerline and calculating its intersection with the vessel surface. Curvature was approximated by calculating Euclidean distances between selected centerline sampling points. Preliminary analyses demonstrated that additional geometric descriptors, including minimal inscribable angle, global curvature, and torsion, did not contribute significantly to classification performance and were therefore excluded.

Spatial normalization was required to ensure that geometric features were extracted from anatomically corresponding regions across patients. Three approaches were evaluated for identifying the center point of the carotid siphon: (1) selection of the centerline point with the maximum anterior patient coordinate, (2) identification of the midpoint of the minimum inscribable angle along the centerline, and (3) interactive selection by the operating neurosurgeon. The first two approaches enabled fully automated processing, whereas the third provided expert-guided correction when necessary.

**Figure 3:**
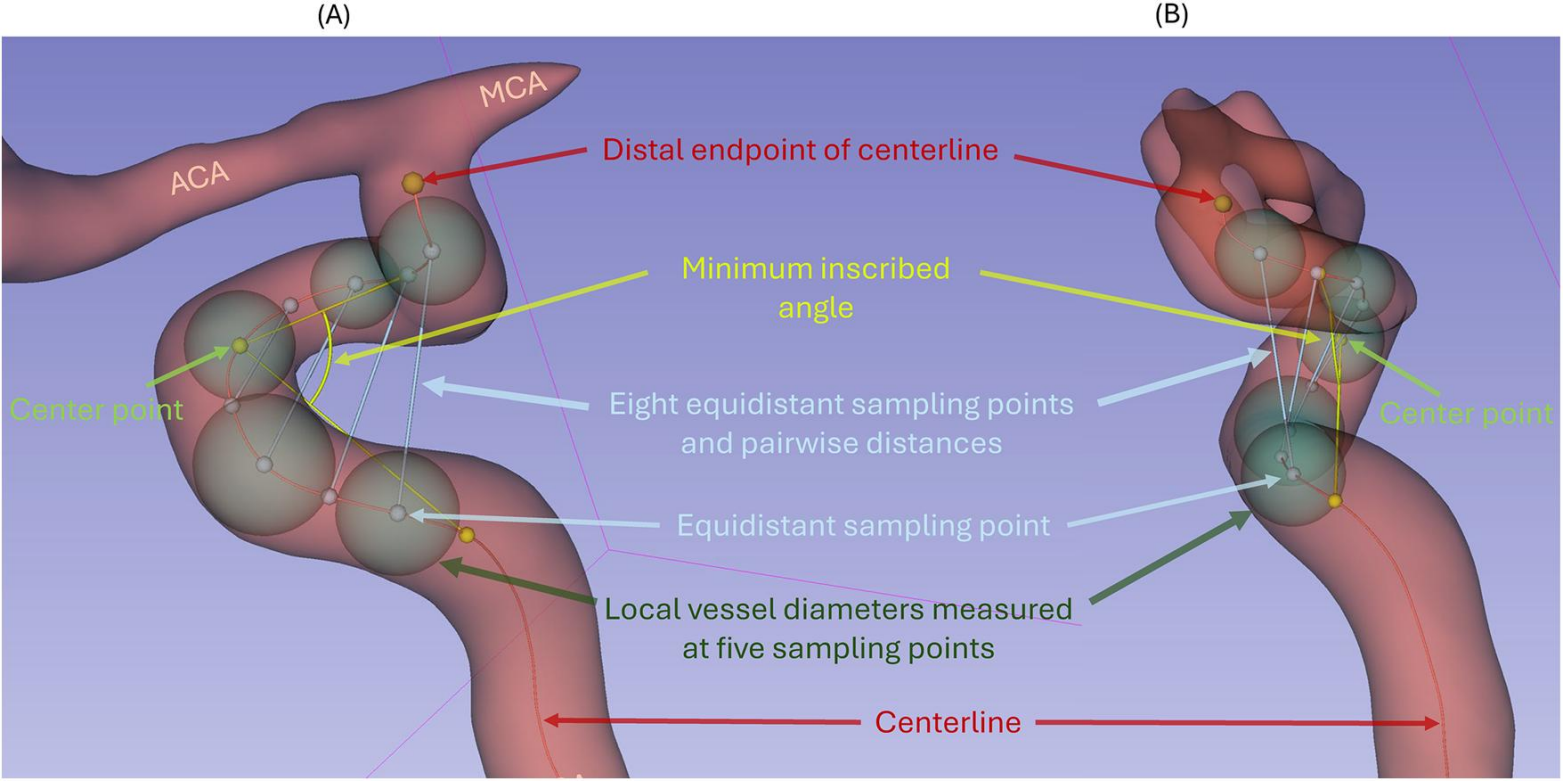
**(A,B)** Vessel centerline, cross-sectional area, and distance measurements for curvature approximation from two views. Sampling points along the vessel centerline are shown as yellow spheres. Green spheres indicate the locations where cross-sectional area was computed; spheres are displayed only for visualization, while classification was based on the calculated cross-sectional area values. Yellow lines represent the distances used for curvature approximation. Panels **A** and **B** show the same vessel segment from two orthogonal views, with panel **B** rotated approximately 90° relative to panel **A**.

After identification of the siphon center point, equidistant sampling points were selected along the centerline in both directions. Five sampling points were used for vessel cross-sectional area measurements, while eight symmetrically distributed sampling points were used for curvature approximation, resulting in four pairwise distance measurements (Figure 3). The distance between adjacent sampling points was set to 0.25 mm.

Additional derived features, such as normalized distances and differences between consecutive cross-sectional areas, were considered during preliminary analyses. However, because of the limited number of studies and the risk of overfitting, only the raw geometric measurements were retained. Furthermore, absolute geometric scale was considered clinically relevant in the carotid siphon. For example, a 1 mm luminal narrowing is substantially more critical in a 2.5 mm artery than in a 4 mm vessel, and the absolute length of a vascular bend directly affects catheter “stiffness” requirements and device “pushability”.

Using the selected nine geometric attributes and the procedural ground-truth labels, automated classifier and hyperparameter optimization were performed in the Weka environment using the Auto-WEKA classifier module with 10-fold cross-validation (24). The optimization process was allowed to run for 10 h in order to identify the best-performing classification pipeline.

The optimal model consisted of a logistic regression classifier with ridge regularization and correlation-based feature subset (CFS) selection using the GreedyStepwise search strategy. Unlike more complex nonlinear machine-learning approaches, the final classifier was based on nine explicitly defined geometric features, allowing the relationship between the measured vascular characteristics and the predicted procedural classes to remain directly interpretable. The selected model components and their purposes are summarized in Table 4.

**Table 4:** Components and parameters of the optimal classification pipeline.

Component	Choice	Purpose
Model	Logistic regression	Predicts class probabilities using a linear combination of features
Regularization	Ridge (*R* ≈ 0.106)	Prevents the model from becoming too complex/overfitted
Selection	CFS + GreedyStepwise	Removes redundant or irrelevant features to improve speed and accuracy

By default, the classifier assigned each case to the most probable procedural category while also providing class-probability estimates reflecting prediction confidence. The probabilities summed to 1.0 and were interpreted as relative confidence values. To further characterize prediction reliability, a confidence criterion was defined. First, the highest and second-highest class probabilities were identified. Predictions were accepted as high-confidence decisions only when the second-highest class probability was less than 60% of the highest class probability. This confidence estimate is generated directly by the trained classifier for each new case and therefore remains available during prospective clinical use. As with any probabilistic classifier, high-confidence predictions may still occasionally be incorrect, but the confidence criterion provides an estimate of prediction certainty rather than a guarantee of correctness.

## Results

**Figure 4:**
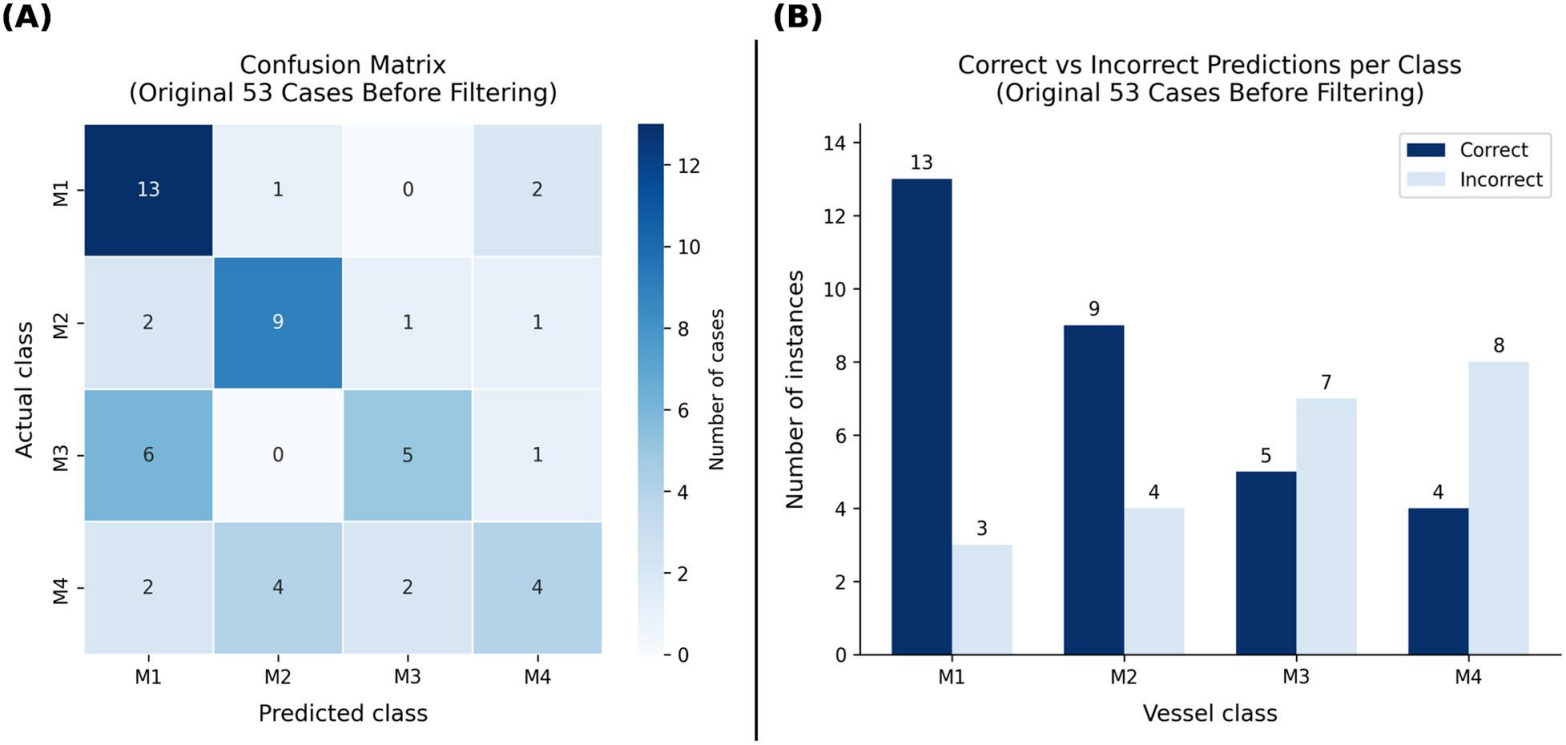
Classification performance in the full 53-case dataset before confidence-based filtering. (A) Confusion matrix showing the relationship between actual procedural device-escalation class and model-predicted class. Rows indicate the actual class, and columns indicate the predicted class. The full dataset shows substantial interclass overlap, particularly for M3 and M4. Several M3 cases were classified as M1, suggesting that some anatomically favorable-appearing vessels nevertheless required more advanced procedural support. M4 predictions were dispersed across multiple classes, indicating limited separability of the highest-complexity category without confidence-based filtering. (B) Bar chart summarizing the number of correct and incorrect predictions for each procedural device-escalation class. Correct classifications predominated in M1 and M2, whereas prediction accuracy decreased in the higher-complexity classes. M3 and M4 showed frequent misclassification, suggesting that advanced device-support requirements may depend on additional anatomical, device-related, or procedural factors not fully captured by the selected geometric descriptors.

The confusion matrix generated from the full dataset (Figure 4A) revealed substantial interclass confusion, particularly between M3 and M1. Half of the M3 cases (6/12) were misclassified as M1, suggesting that geometric descriptors alone may not fully capture procedural complexity in certain anatomically deceptive cases. Predictions for M4 were distributed across all categories, indicating limited separability of the highest-complexity class in the absence of confidence-based filtering.

Figure 4B shows the distribution of correct and incorrect predictions across the four procedural classes. Correct classifications predominated in M1 and M2, indicating better model performance in lower-complexity cases. In contrast, prediction accuracy decreased for M3 and M4, where incorrect classifications were frequent. This suggests that higher-complexity procedures may involve additional anatomical or technical factors not fully represented by the current morphometric feature set.

After applying the confidence-based decision criterion defined in the Methods section, 32 of the original 53 cases were retained as high-confidence predictions. This filtering increased overall classification accuracy from 58.5% in the full dataset to 75.0% in the high-confidence subset, while the kappa statistic increased from 0.40 to 0.65. The weighted F-measure in the high-confidence subset was 0.73 (Table 5).

**Table 5:** Comparison of classification performance before and after confidence-based filtering.

Metric	Original (53 cases)	Filtered (32 cases)	Improvement
Accuracy	58.5%	75.0%	+16.5%
Kappa statistic	0.40 (fair)	0.65 (substantial)	+0.25
M1 Precision	68.4%	68.8%	+0.4% (stable)
M2 Precision	64.3%	77.8%	+13.5%
M3 Precision	62.5%	100.0%	+37.5%
F-Measure (Wtd.)	0.58	0.73	+0.15

**Figure 5:**
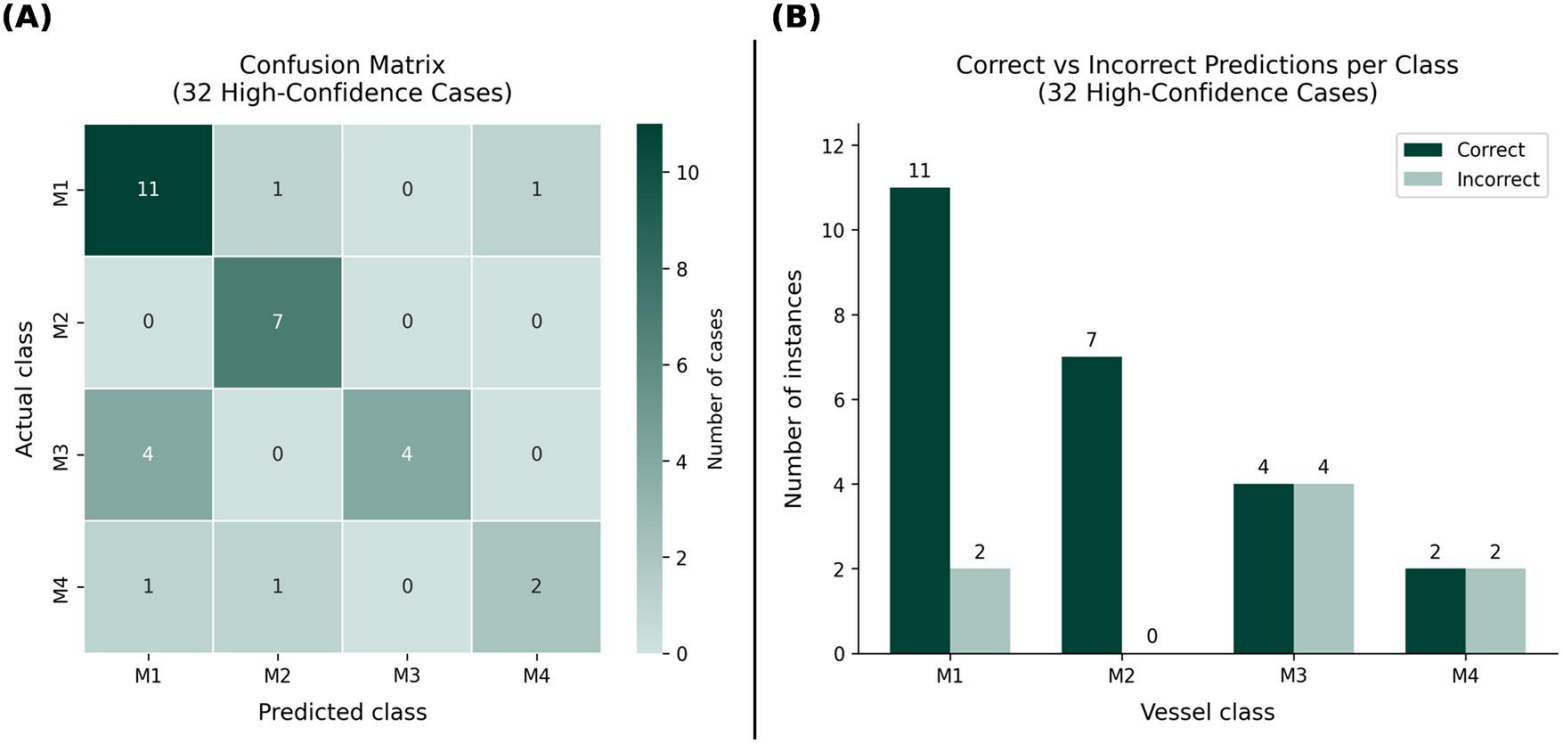
Classification performance in the 32 high-confidence cases after confidence-based filtering. (A) Confusion matrix showing classification performance after applying the confidence-based decision criterion. Cases were retained only when the second-highest class probability was less than 60% of the highest class probability. This filtering reduced the dataset from 53 to 32 cases and improved the interpretability of the classification results. The model showed clear identification of M2 cases and high precision for M3 predictions, although residual misclassification remained, particularly between M1 and M3. (B) Bar chart summarizing the number of correct and incorrect predictions across procedural device-escalation classes in the 32 high-confidence cases. After filtering, correct predictions clearly predominated in M1 and M2. In contrast, M3 and M4 remained more difficult to classify, with correct and incorrect predictions occurring at similar frequencies. These findings suggest that confidence-based filtering improves identification of well-defined anatomical patterns but does not fully resolve ambiguity in the highest-complexity procedural categories.

The confusion matrix for the high-confidence cases is shown in Figure 5A. The model showed the strongest performance in the lower- and intermediate-complexity categories. M1 was correctly classified in 11 of 13 cases, with two misclassifications assigned to M2 and M4, respectively. All M2 cases were correctly classified, corresponding to 100% recall for this class.

For M3, 4 of 8 cases were correctly classified, while the remaining 4 cases were predicted as M1. However, all cases predicted as M3 were true M3 cases, corresponding to 100% precision for the M3 class. M4 remained less consistently classified, with 2 of 4 cases correctly predicted, while the remaining cases were assigned to M1 and M2.

The distribution of correct and incorrect predictions by procedural class is shown in Figure 5B. Correct classifications predominated in M1 and M2, whereas M3 and M4 showed equal numbers of correct and incorrect predictions after confidence-based filtering.

Table 6 provides an interpretation of the classification errors observed in the full 53-case dataset and helps distinguish between high-confidence anatomical patterns and more ambiguous cases. Based on the confidence-based filtering approach, the original cohort can be conceptually divided into two subsets.

**Table 6:** Interpretation of classification errors and ambiguous cases in the full 53-case dataset.

Observation	Sequential strategy interpretation
M3 predicted as M1	These cases may represent anatomically deceptive vessels. Although classified clinically as M3 because advanced support was ultimately required, their measured geometric features resembled lower-complexity M1 cases. This suggests that selected morphometric parameters alone may not fully capture all factors contributing to procedural escalation.
Stable classification of M2 in the high-confidence subset	In the high-confidence subset, M2 was identified with particularly high stability. This indicates a relatively distinct transition between cases manageable with direct or minimal support and those requiring standard coaxial/triaxial assistance.
M4 as the highest-escalation category	M4 represents the final escalation level, used only after lower-support strategies have failed. Therefore, this class is likely influenced by the greatest number of anatomical, mechanical, and procedural factors, making it the most difficult category to predict using geometric features alone.

The first subset consists of high-confidence cases (*n* = 32), in which the extracted geometric features showed a strong association with the procedural device-escalation category. These cases likely represent vessels with more clearly defined anatomical characteristics, such as relatively favorable or markedly complex siphon geometry, allowing the model to assign the procedural class with greater confidence.

The second subset consists of low-confidence or ambiguous cases (*n* = 21), in which the anatomical features did not provide a sufficiently distinct classification signal. These cases may represent borderline vascular geometries, where procedural escalation was influenced not only by measurable vessel morphology but also by additional factors not captured in the current model, such as vessel wall calcification, catheter-vessel interaction, vascular compliance, operator preference, or intra-procedural decision-making.

Thus, the confidence-based separation suggests that CTA-derived morphometric features may reliably predict device requirements in anatomically well-defined cases, whereas borderline cases may require additional clinical, anatomical, or procedural variables to improve prediction accuracy.

## Discussion

### Interpretation of the sequential device-escalation strategy

To our knowledge, this is the first study to correlate CTA-derived three-dimensional carotid siphon morphology with hierarchical device escalation during mechanical thrombectomy. Previous studies have examined the relationship between carotid siphon anatomy and endovascular device navigation in different contexts, including aspiration catheter deliverability (25), virtual thrombectomy simulations (19), and the broader influence of vascular tortuosity on catheter navigability and device compatibility (3–5, 8, 9). However, these studies have generally relied on simplified anatomical descriptors, experimental or computational models, or device-specific endpoints rather than directly linking patient-specific three-dimensional carotid siphon geometry to stepwise device escalation during real-world thrombectomy procedures.

Bridio et al. used patient-specific vascular models for virtual thrombectomy simulation and showed that internal carotid artery morphology, including curvature characteristics of the carotid siphon, may influence simulated thrombectomy success (19). This represented an important conceptual advance, but the study was based on a limited number of simulated cases and did not evaluate procedural device escalation in clinical practice. Similarly, Kuriyama et al. investigated anatomical factors affecting Penumbra catheter navigation through the carotid siphon and found that a smaller carotid siphon radius was associated with interruption of catheter navigation, supporting the concept that local siphon geometry can influence device deliverability (25).

The present study represents a multidisciplinary collaboration between the Department of Neurosurgery, the Department of Medical Physics and Informatics, and the Department of Image Processing and Computer Graphics at the University of Szeged. Mechanical thrombectomy procedures and procedural data were collected in the neurosurgical clinical setting, while three-dimensional CTA processing, morphometric analysis, and computational modeling were performed using dedicated image-processing and machine-learning workflows.

At the Department of Neurosurgery, catheter navigation follows a hierarchical escalation strategy in which progressively more supportive devices are introduced only when simpler approaches fail (1, 2). Consequently, the recorded intervention class does not merely reflect operator preference but also represents a practical threshold of anatomical resistance encountered during the procedure. Within this framework, successful direct aspiration without additional support (M0 success) reflects very favorable anatomy; success with microwire assistance (M0 failure/M1 success) indicates mild navigational resistance; success only after coaxial or triaxial support (M1 failure/M2 success) indicates moderate tortuosity; the need for reinforced high-support navigation (M2 failure/M3 success) reflects severe tortuosity; and stent retriever-assisted anchoring (M3 failure/M4 success) represents the highest level of anatomical difficulty. This sequential structure provides a clinically meaningful opportunity to interpret device escalation as a surrogate marker of anatomical resistance during catheter navigation.

### Relationship between carotid siphon geometry and device escalation

The present study demonstrates that geometric features derived from CTA-based three-dimensional reconstruction of the carotid siphon contain clinically relevant information regarding catheter navigation difficulty. The objective was not to introduce a new categorical anatomical classification of carotid siphon morphology, but to determine whether continuous quantitative descriptors of vessel caliber and curvature contain sufficient information to predict procedural device-support requirements without imposing predefined morphological categories. When all 53 cases were included, the classification model achieved moderate predictive performance. Inspection of the full-dataset confusion matrix (Figure 4A) revealed that a substantial proportion of misclassifications occurred in anatomically borderline cases, in which catheter advancement may have been possible with simpler devices but required increased procedural persistence, operator adaptation, or subtle changes in device handling.

A key source of this ambiguity is the overlap of geometric features at escalation thresholds. Vessels in which a simpler device barely succeeded may produce morphometric measurements that closely resemble vessels in which the same device failed and escalation was required. In these situations, similar geometric signals may correspond to different recorded procedural outcomes, contributing to the prediction dispersion observed in the full-dataset confusion matrix. This represents an inherent limitation of retrospective procedural classification, because the final device class may reflect both anatomical constraints and individual intra-procedural decision-making.

This interpretation is consistent with prior work showing that unfavorable vascular anatomy is associated with increased procedural difficulty and delayed reperfusion during endovascular stroke therapy. Ribo et al. reported that difficult catheter access to the occluded vessel was associated with worse clinical outcome (13), while other studies and reviews have emphasized that tortuous cervical or intracranial anatomy can prolong access time, increase the need for bailout strategies, and complicate device delivery; experimental and computational studies further suggest that device performance in tortuous anatomy is influenced by retrieval forces, frictional interaction, and procedural parameters such as catheter position (26–28).

### High-confidence anatomical patterns

Confidence-based filtering improved model performance and helped identify a subset of cases in which carotid siphon morphology provided a clearer predictive signal. The increase in accuracy from 58.5% to 75.0%, together with the improvement in kappa from 0.40 to 0.65, suggests that CTA-derived cross-sectional and curvature-based features can capture clinically meaningful information in anatomically well-defined cases.

The particularly stable classification of M2 suggests that moderate-complexity anatomy requiring standard coaxial or triaxial support may represent a relatively distinct procedural category. In these cases, geometric features appear to provide sufficient information to distinguish vessels requiring more than minimal assistance but not the highest levels of support.

The 100% precision observed for M3 indicates that, when the model identified a case as requiring reinforced high-support navigation, this prediction was specific for the M3 category. However, only half of the actual M3 cases were correctly classified, with the remaining cases predicted as M1. This discrepancy suggests that some clinically difficult vessels may appear geometrically favorable according to the selected morphometric descriptors. In such cases, procedural difficulty may have been driven by factors not captured by the current model, such as catheter–vessel interaction, arterial compliance, plaque burden, calcification, device behavior, or operator-dependent technique.

M4 remained the most difficult category to distinguish, even after confidence-based filtering. This is plausible because anchor-based escalation represents not only severe luminal geometry but also failure of preceding support strategies, where distal support, device length, catheter stability, and operator-specific bailout techniques may all contribute (29). This may reflect the fact that the highest escalation level is not determined by a single geometric feature, but rather by a combination of anatomical, mechanical, and procedural factors. Therefore, while carotid siphon morphology provides useful preprocedural information, prediction of the most complex navigation scenarios will likely require additional variables beyond luminal geometry alone.

Overall, these findings support the use of confidence-gated classification as a practical strategy. This concept is aligned with recent trends toward patient-specific and uncertainty-aware thrombectomy planning, in which imaging-derived anatomical information and model confidence may help guide device preparation while preserving expert procedural judgment (30–32). High-confidence predictions may help identify cases in which anatomy provides a strong procedural signal, whereas low-confidence or discordant cases may indicate borderline anatomy requiring procedural flexibility and early preparation for escalation.

### Borderline cases and procedural variability

The 21 cases excluded by the confidence filter represent a clinically important intermediate group. These vessels likely exhibit borderline anatomical configurations in which catheter navigation success depends not only on vascular geometry but also on additional procedural and mechanical factors. These may include catheter design, device stiffness, vessel wall compliance, calcification, local plaque morphology, support from proximal access devices, frictional device–vessel interaction, and operator experience (13, 27, 28, 33, 34). This interpretation is consistent with prior work showing that vascular tortuosity and difficult catheter access influence thrombectomy performance, while experimental and *in silico* studies indicate that device-vessel and device-thrombus interactions are governed by multiple mechanical factors beyond geometry alone (19, 35).

Model uncertainty should therefore not necessarily be interpreted as model failure. Rather, uncertainty may identify cases in which anatomy alone is insufficient to determine the optimal procedural strategy. This concept is consistent with uncertainty-aware medical imaging models, where low-confidence predictions may be withheld from automated classification and referred for expert review (30). In the present setting, a confidence-gated decision-support tool could alert the operator that procedural flexibility, alternative devices, or early preparation for escalation may be required.

These observations highlight the complex and multifactorial nature of endovascular navigation during mechanical thrombectomy. A geometry-based model, even when derived from three-dimensional reconstruction, is unlikely to fully explain procedural difficulty in all cases. Nevertheless, the ability to identify high-confidence anatomical patterns may still provide useful preprocedural information for a clinically meaningful subset of patients. This is supported by patient-specific simulation studies showing that local vascular morphology influences thrombectomy behavior, but that simplified or global geometric descriptors alone may not fully capture procedural outcome (19).

### Clinical implications

Despite these limitations, the present findings suggest that automated analysis of carotid siphon geometry may provide clinically useful preprocedural information. Such an approach could help anticipate the need for advanced device support, support device preparation before groin puncture, and reduce delays associated with repeated unsuccessful navigation attempts or late device escalation. Earlier anticipation of the required support strategy may reduce unnecessary device exchanges and facilitate more efficient procedural preparation. Although puncture-to-recanalization time was not evaluated in the present study, this represents an important endpoint for future prospective validation. This is clinically important because faster reperfusion is strongly associated with improved functional outcomes after mechanical thrombectomy (1, 2). The HERMES collaboration demonstrated a time-dependent decline in benefit from endovascular therapy, reinforcing the importance of minimizing delays throughout the thrombectomy workflow (2, 36).

The proposed confidence-gating framework may offer a practical implementation pathway. In cases where the model provides a high-confidence prediction, the output could inform device preparation and procedural planning. In cases where the model returns a low-confidence prediction, the uncertainty itself may be clinically informative by indicating that the anatomy is borderline and that additional non-geometric factors are likely to influence procedural success.

Importantly, such a tool would not be intended to replace operator judgment. Instead, CTA-derived morphometric analysis could complement existing preprocedural assessment by providing objective, reproducible information about carotid siphon geometry. With further validation, this type of analysis could contribute to patient-specific planning in endovascular stroke treatment, particularly in centers where device choice, preparation time, and resource utilization are important procedural considerations.

### Limitations

Several limitations should be acknowledged. First, the sample size was relatively small, reflecting the single-center and retrospective design. Although the results demonstrate promising associations between carotid siphon geometry and procedural device escalation, larger multicenter datasets will be necessary to confirm the robustness and generalizability of the findings.

Second, procedural difficulty was inferred from the device-escalation level ultimately required during intervention. Although the hierarchical escalation protocol reduces subjectivity, the final device category may still be influenced by operator experience, procedural strategy, device availability, and intra-procedural decision-making.

Third, the model used a deliberately limited set of geometric parameters because of the relatively small dataset. While this reduced the risk of overfitting, additional morphological descriptors (such as curvature profiles, torsion, diameter variation, bend length, and local angulation) as well as non-geometric factors including calcification, plaque characteristics, vessel stiffness, catheter design, and guide-catheter support may influence navigation difficulty and should be considered in future multimodal models.

The present model was specifically designed to predict the level of device support required for catheter navigation through the carotid siphon prior to thrombus engagement. Consequently, outcomes such as the number of thrombectomy passes, reperfusion grade (mTICI), or total procedure duration were beyond the scope of the current study, as these depend on additional factors including thrombus location, clot burden, clot composition, thrombectomy technique, and operator strategy. Future studies integrating both vascular and thrombus-related characteristics may extend the proposed framework toward prediction of these clinically relevant endpoints.

Finally, the present analysis was based on retrospective imaging and procedural data from a single institution. Prospective validation will be required to determine whether CTA-derived carotid siphon morphometry can improve procedural planning, reduce navigation time, or decrease unnecessary device escalation in routine clinical practice.

## Conclusion

This study demonstrates that CTA-derived three-dimensional morphometric analysis of the carotid siphon provides objective anatomical information that correlates with hierarchical device escalation during mechanical thrombectomy. The findings suggest that selected geometric features of the carotid siphon can serve as surrogate markers of anatomical resistance encountered during catheter navigation.

Using a confidence-gated classification framework, we showed that cases with distinct morphological patterns allow more reliable prediction of the device support required for successful navigation. In contrast, borderline cases remain influenced by multifactorial procedural variables, including catheter-vessel interaction, vessel wall properties, device characteristics, and operator-dependent technique.

These results support the potential integration of automated vascular characterization into preprocedural decision-support systems for endovascular stroke treatment. By anticipating the need for advanced instrumentation, such tools may help streamline procedural planning, reduce avoidable delays, and optimize the use of specialized devices. Larger prospective multicenter studies are warranted to validate these findings, refine predictive models, and define the clinical role of CTA-based carotid siphon morphometry in patient-specific thrombectomy planning.

## Data Availability

The raw data supporting the conclusions of this article will be made available by the authors, without undue reservation.
